# Supplementary material for: Heterogeneity of Integrin αIIbβ3 Function in Pediatric Immune Thrombocytopenia Revealed by Continuous Flow Cytometry Analysis
Source: Int J Mol Sci. 2020 Apr 25;21(9):3035. doi: 10.3390/ijms21093035 (PMC7246588; doi:10.3390/ijms21093035)
Supplement: Supplementary file 1 [file ijms-21-03035-s001.pdf]

Supplementary Materials

A heterogeneity of integrin $\alpha_{IIb}\beta_3$ function in pediatric immune thrombocytopenia revealed by continuous flow cytometry analysis

Alexey A. Martyanov[1,2,3,4], Daria S. Morozova[5], Maria A. Sorokina[1], Aleksandra A. Filkova[1,2,4], Daria V. Fedorova[1], Selima S. Uzueva[1], Elena V. Suntsova[1], Galina A. Novichkova[1], Pavel A. Zharkov[1], Mikhail A. Panteleev[1,2,4,6] and Anastasia N. Sveshnikova[1,2,4,7,*]

[1] National Medical Research Center of Pediatric Hematology, Oncology and Immunology named after Dmitry Rogachev, 1 Samory Mashela St, Moscow, 117198, Russia
[2] Center for Theoretical Problems of Physico-Chemical Pharmacology, Russian Academy of Sciences, 30 Srednyaya Kalitnikovskaya str., Moscow, 109029, Russia
[3] Institute for Biochemical Physics (IBCP), Russian Academy of Sciences (RAS), Russian Federation, Moscow, Kosyigina 4; 119334, Russia
[4] Faculty of Physics, Lomonosov Moscow State University, 1/2 Leninskie gory, Moscow, 119991, Russia
[5] Faculty of Basic Medicine, Lomonosov Moscow State University, 27/1 Lomonosovsky av., Moscow, 119991, Russia
[6] Faculty of Biological and Medical Physics, Moscow Institute of Physics and Technology, 9 Institutskii per., Dolgoprudnyi, 141700, Russia
[7] Department of Normal Physiology, Sechenov First Moscow State Medical University, 8/2 Trubetskaya St., Moscow, 119991, Russia
***** Correspondence: a.sveshnikova@physics.msu.ru

Keywords: platelet; integrin αIIbβ3; calcium signaling; immune thrombocytopenia; blood platelet disorders; flow cytometry; computational modeling.

Supplementary Tables.. 2

Supplementary Figures.. 5

Computational model development and analysis 10

References 13

Supplementary Tables

Table S1. Characteristics of ITP patients

Gender	Age	Plt count	Bleeding	Therapy at the time of the analysis	History of therapy	Type of ITP	Subgroup
m	5	29	1	IVIG + dexamethasone	IVIG, glucocorticoids	Persistent	HFB
m	9	25	2	None	IVIG, IFN alpha2b, glucocorticoids	Chronic	HFB
m	8	150	1	None	prednisolone	Chronic	HFB
f	12	36	2	Romiplastim	IFN alpha2b, romiplastim	Persistent	HFB
f	9	209	0	None	IVIG. glucocorticoids, IFN alpha2b	Chronic	HFB
m	11	32	0	None	IVIG	Chronic	HFB
m	15	86	0	None	None	Chronic	HFB
m	2	74	1	None	IVIG	Acute	HFB
m	5	14	2	IVIG	IVIG, glucocorticoids, IFN alpha2b	Chronic	HFB
f	15	72	0	None	None	Chronic	HFB
f	15	128	0	None	None	Persistent	HFB
m	2	67	1	None	prednisolone, IVIG	Chronic	LFB
m	10	93	0	IFN alpha2b 1000000 ME once a week	IVIG, methylprednisolone, dexamethasone, IFN alpha2b	Chronic	LFB
m	16	31	1	None	glucocorticoids, IFN alpha2b	Chronic	LFB
f	7	58	2	None	IVIG, glucocorticoids	Persistent	LFB
f	4	41	1	None	glucocorticoids, IVIG	Chronic	LFB
f	14	8	1	None	etamsylate, tranexamic acid, glucocorticoids	Chronic	LFB
f	10	27	1	prednisolone 25 mg/day	glucocorticoids, IVIG	Persistent	LFB
m	7	36	0	None	IVIG, glucocorticoids	Persistent	LFB
f	10	67	2	None	IVIG, glucocorticoids	Chronic	LFB
m	15	72	1	None	IVIG, glucocorticoids	Acute	LFB
f	2	342	0	None	IVIG	Persistent	LFB
m	2	109	1	None	IVIG	Persistent	LFB
f	6	25	1	None	prednisolone, IVIG	Persistent	LFB
f	7	70	0	None	IVIG	Persistent	LFB
m	8	41	2	None	glucocorticoids, IVIG, romiplostim, rituximab, splenectomy	TPO[1]	LFB
f	11	254	0	IFN alpha2b 1000000 ME/week	prednisolone, dexamethasone, IVIG, IFN alpha2b	TPO[1]	LFB
f	7	327	0	eltrombopag, IVIG	IVIG, glucocorticoids, eltrombopag	TPO[1]	LFB

m	8	41	2	None	glucocorticoids, IVIG, romiplostim, rituximab, splenectomy	TPO[1]	LFB
f	9	155	1	None	IVIG	Chronic	LFB
m	6	9	1	romiplostim 5 mkg/kg №2	IVIG, glucocorticoids, romiplostim	Persistent	LFB
m	8	19	1	None	glucocorticoids	Persistent	LFB
f	2	19	1	romiplostim	IVIG, glucocorticoids, romiplostim	TPO[1]	LFB
m	5	151	2	None	IVIG, glucocorticoids	Chronic	NC
m	17	103	2	None	glucocorticoids, IVIG, splenectomy	Chronic	NC
f	12	174	0	glucocorticoids, IVIG	etamsylate, IVIG, glucocortiicoids	Acute	NC

[1] Agonists of thrombopoietin receptor in the medical history

Table S1a. Characteristics of GT patients

Gender	Age	Plt count	Therapy at the time of the analysis	History of therapy
f	31	29	None	None
f	2	No data	Maltofer, folic acid	Maltofer, folic acid
f	7	264	Maltofer, folic acid	Maltofer, folic acid
m	5	303	Eptacog alpha	Eptacog alpha
f	7	344	None	None
f	6	No data	Tranexam, Maltofer	Tranexam, Maltofer
f	10	180	Tranexam, Ferrum Lek	Tranexam, Ferrum Lek
f	No data			

Table S2. Reactions and parameters for the integrin activation module

Name	Reaction	Flux, compartment	Parameters	Reference
Activation of PI3Kγ	$\mathrm{PI3K\gamma} \leftrightarrow \mathrm{PI3K\gamma}^*$	$\frac{k_1 \cdot V_{cyt}[G\beta\gamma][\mathrm{PI3K\gamma}]}{(L_1+[G\beta\gamma])\cdot S_{pm}} + \frac{k_2 \cdot V_{cyt}[\mathrm{PI3K\gamma}]}{S_{pm}} - k_3 \cdot [\mathrm{PI3K\gamma}^*]$, PM	$k_1 = 0.001\ s^{-1}, k_2 = 0.001\ s^{-1}$, $k_4 = 0.03\ s^{-1}$, $L_1 = 2 \cdot 10^{-18}\ \frac{\mu mol}{\mu m^2}$	Adapted from [1,2]
PI3Kγ	$PIP_2 \rightarrow PIP_3$	$\frac{k[PIP_2][\mathrm{PI3K\gamma}^*]}{K_M+[PIP_2]}$, PM	$k = 100\ s^{-1}$, $K_M = 6.4 \cdot 10^{-16}\ \frac{\mu mol}{\mu m^2}$	[3,4]
	$PIP_{1(4)} \rightarrow PIP_{2(3,4)}$	$\frac{k[PIP_{1(4)}][\mathrm{PI3K\gamma}^*]}{K_M+[PIP_{1(4)}]}$, PM		
PTEN	$PIP_3 \rightarrow PIP_2$	$\frac{V[PIP_3]^2}{K^2+[PIP_3]^2}$, PM	$V = 2 \cdot 10^{-20}\ \frac{\mu mol}{\mu m^2} \cdot s^{-1}$, $K = 2 \cdot 10^{-17}\ \frac{\mu mol}{\mu m^2}$	[3,5]
	$PIP_{2(3,4)} \rightarrow PIP_{1(4)}$	$\frac{V[PIP_{2(3,4)}]^2}{K^2+[PIP_{2(3,4)}]^2}$, PM		
SHIP	$PIP_3 \rightarrow PIP_{2(3,4)}$	$\frac{V[PIP_3]}{K+[PIP_3]}$, PM	$V = 5.8 \cdot 10^{-18}\ \frac{\mu mol}{\mu m^2} \cdot s^{-1}$, $K = 1 \cdot 10^{-17}\ \frac{\mu mol}{\mu m^2}$	[3,6]
	$PIP_2 \rightarrow PIP_{1(4)}$	$\frac{V[PIP_2]}{K+[PIP_2]}$, PM		
Activation of CalDAGGEFI	$CDGEF1 + Ca^{2+}_{cyt} \leftrightarrow\leftrightarrow CDGEF1\_Ca$	$k_1[Ca^{2+}_{cyt}][CDGEF1] - k_{-1}[CDGEF1\_Ca]$, cytosol	$k_1 = 1.6(\mu M \cdot s)^{-1}$, $k_{-1} = 0.01\ s^{-1}$	[3,7,8], adjusted
	$CDGEF1\_Ca + Ca^{2+}_{cyt} \leftrightarrow \leftrightarrow CDGEF1^*$	$k_1[Ca^{2+}_{cyt}][CDGEF1\_Ca]\frac{S_{pm}}{V_{cyt}} - k_{-1}[CDGEF1^*]\frac{S_{pm}}{V_{cyt}}$, cytosol		
RASA3 inhibition	RASA3 + N*PIP3 $\leftrightarrow$ $RASA3i$	$k_1[RASA3][PIP3]^N - k_{-1}[RASA3i]$, PM	$N = 10, k_1 = 1 \cdot 10^{-4}\left(\left(\frac{\mu mol}{\mu m^2} \cdot 10^{17}\right)^N s\right)^{-1}$, $k_{-1} = 6.15 \cdot 10^2\ s^{-1}$	This work
Activation of Rap1	$Rap1GDP \leftrightarrow Rap1 + GDP$	$(k_2 + k_1[CDGEF1^*])[Rap1GDP] - k_{-1}[Rap1][GDP]$, PM	$k_1 = 1.8 \cdot 10^{-15}\left(\frac{\mu mol}{\mu m^2} s\right)^{-1}, k_2 = 1.5 \cdot 10^{-4}\ s^{-1}$, $k_{-1} = 1.5\ (\mu M \cdot s)^{-1}$	[3,9,10], adjusted
	$Rap1GTP \leftrightarrow Rap1 + GTP$	$k_2[Rap1GDP] - k_{-1}[Rap1][GTP]$, PM		
	$Rap1GTP \rightarrow Rap1GDP$	$(k_1 + k_2[RASA3])[Rap1GTP]$, PM	$k_1 = 5 \cdot 10^{-4}\ s^{-1}$, $k_2 = 10^{-17}\left(\frac{\mu mol}{\mu m^2} \cdot s\right)^{-1}$	
Integrin activation	$Int \leftrightarrow Int^*$	$k_1 \frac{[Rap1GTP]^2}{K+[Rap1GTP]^2}[Int] - k_{-1}[Int^*]$, PM	$k_1 = 10 s^{-1}$, $K = 0.2 \cdot 10^{34} \cdot \frac{\mu mol^2}{\mu m^4}$, $k_{-1} = 56\ s^{-1}$	This work
Integrin clustering	$N * Int^* \leftrightarrow C\_Int$	$k_1[Int]^N - k_{-1}[C\_Int]$, PM	$N = 10, k_1 = 0.5\left(\left(\frac{\mu mol}{\mu m^2} \cdot 10^{17}\right)^{N-1} s\right)^{-1}$, $k_{-1} = 1.25 \cdot 10^{-4}\ s^{-1}$	This work

Supplementary Figures

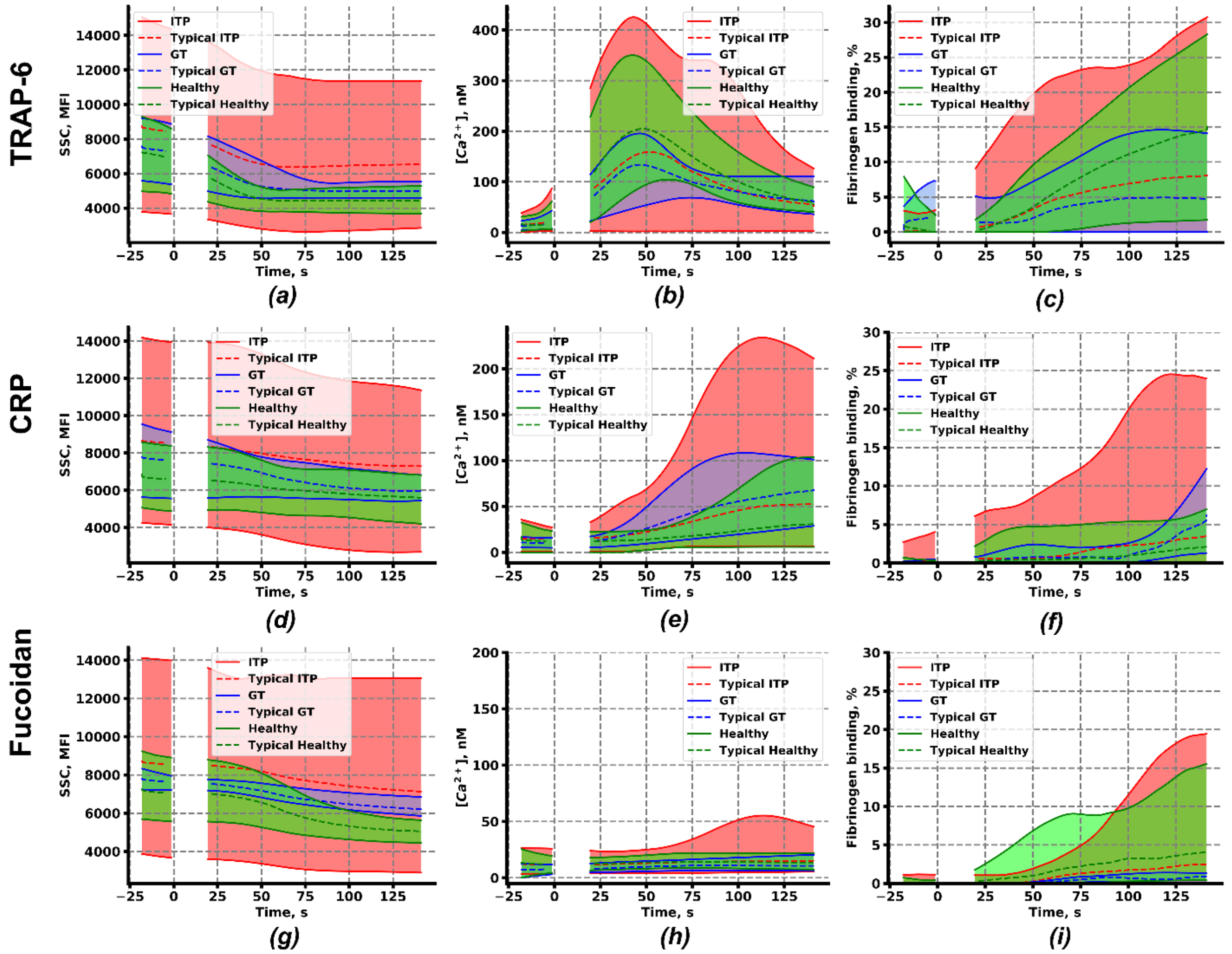

Figure S1. Continuous flow cytometry-based assessment of intracellular calcium concentration, fibrinogen binding and shape change in platelets upon activation with TRAP (a-c), CRP (d-f) and fucoidan (g-i). Envelopes of the mean SSC (left: a,d,g), cytosolic calcium (center: b,e,h) and fibrinogen binding (right: c,f,i) curves of patients with ITP (n = 36, red curves), Glanzmann thrombasthenia (n = 8, blue curves) and healthy donors (n = 10, green curves) upon activation by TRAP (1 μM), CRP (1 μg/ml) or fucoidan (100 μg/ml).

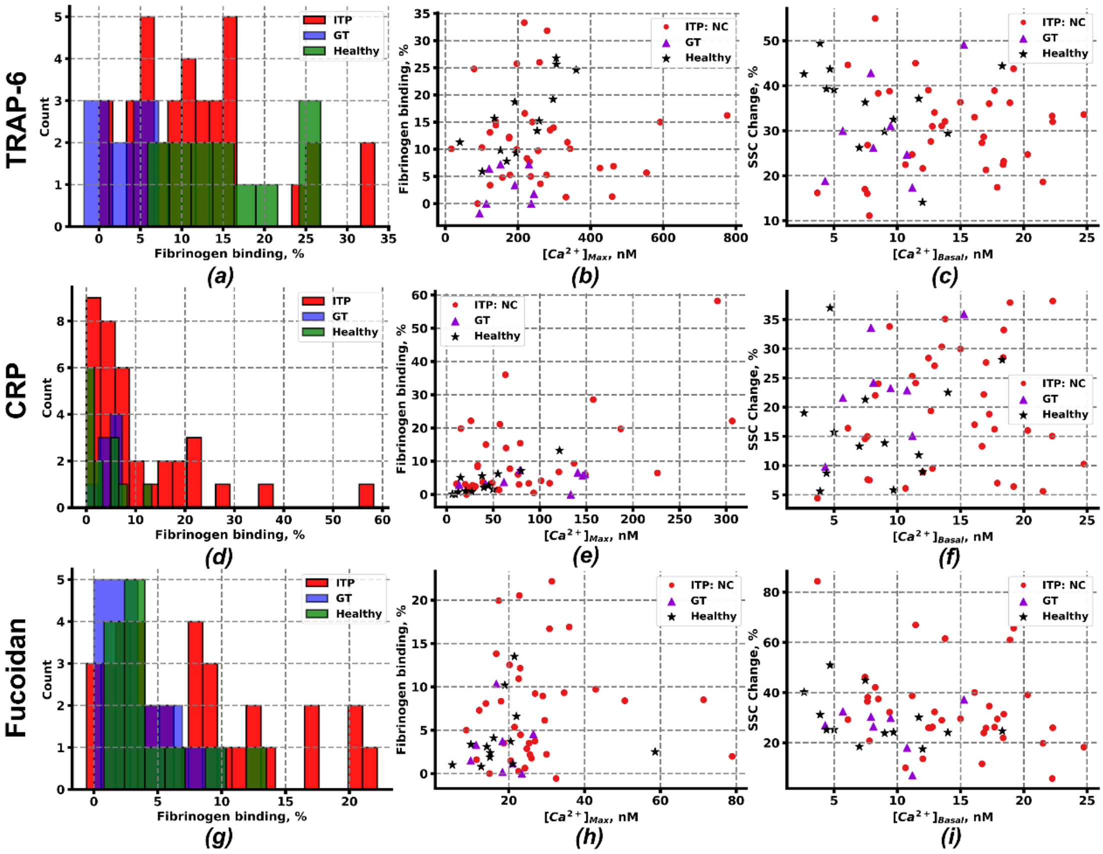

Figure S2. Identification of subgroups of ITP patients for stimulation with TRAP (a-c), CRP (d-f) and fucoidan (g-i). (left: a,d,g) Histograms of fibrinogen binding to platelets of patients with ITP (ITP, red), Glanzmann thrombasthenia (GT, blue) and healthy donors (green). (b,c,e,f,h,i) Cluster analysis of ITP patients by H-DBSCAN clustering algorithm did not reveal subgroups. The same characteristics for GT (purple triangles) and healthy donors (black stars) are given for comparison purposes. Activation by TRAP (1 µM), CRP (1 µg/ml) or fucoidan (100 µg/ml).

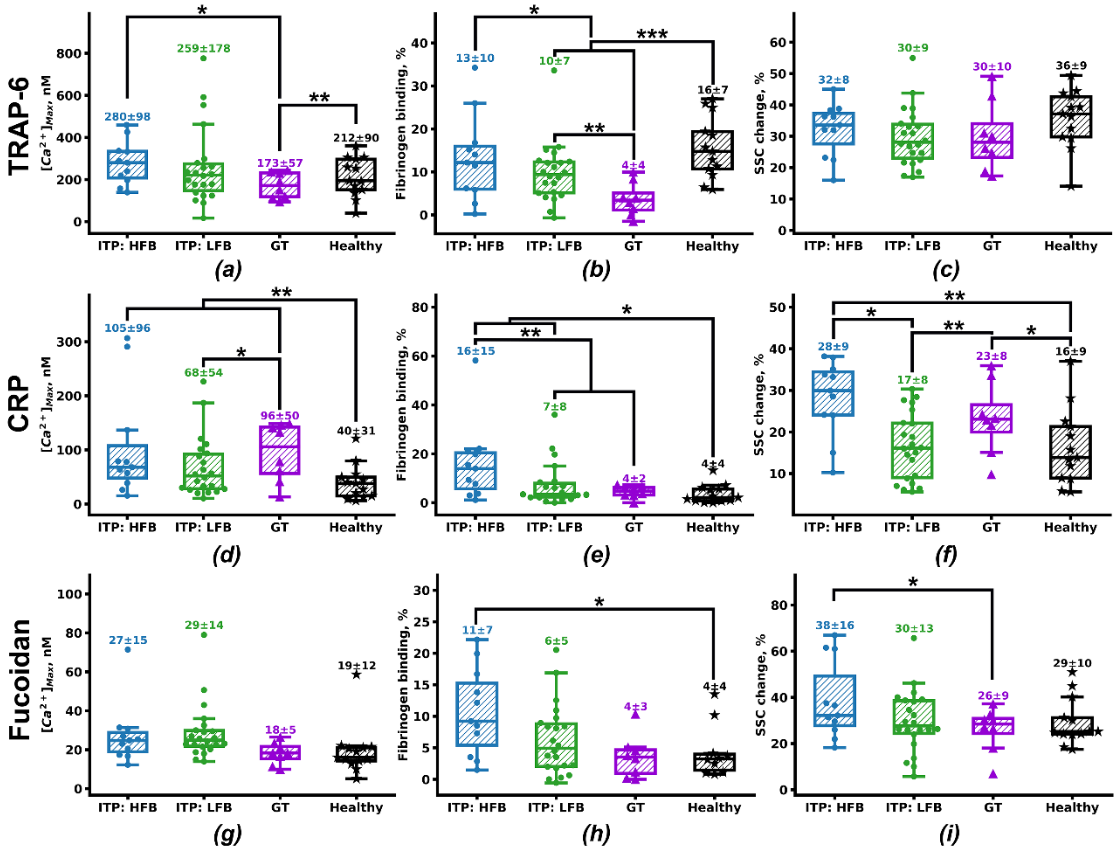

Figure S3. Characterization of HFB and LFB subgroups of ITP patients for stimulation with TRAP (a-c), CRP (d-f) and fucoidan (g-i). Mean values ± SEM for the analyzed groups upon platelet stimulation with TRAP (1 μM), CRP (1 μg/ml) or fucoidan (100 μg/ml) for maximal calcium concentration (a,d,g), fibrinogen binding (b,e,h), and shape change (c,f,i). Statistics was calculated by Mann-Whitney test, * - $p < 0.05$, ** - $p < 0.01$, *** - $p < 0.001$

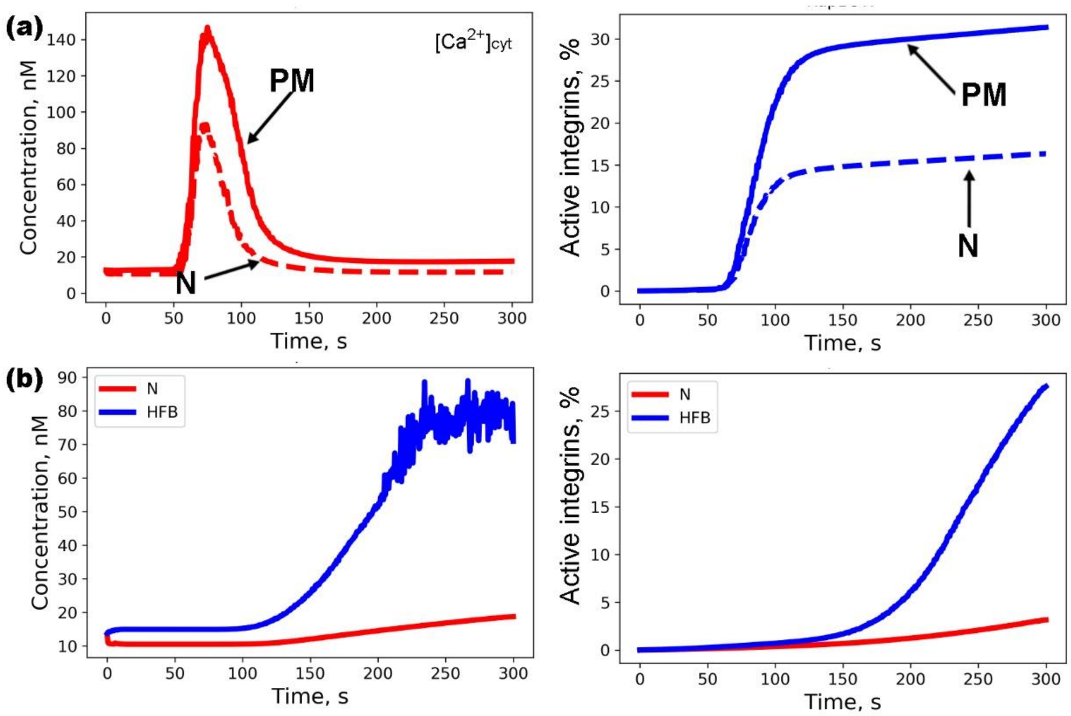

Figure S4. Variation of computational model parameters. (a) Increased cytosolic calcium as a result of platelet increased cell plasma membrane calcium conductivity. Initial IP_3 concentration (not shown) was assumed to be

the same as in the initial model, while higher basal calcium concentration (PM, solid red) was sustained by increased calcium influx through the plasma cell membrane without significant integrin activation (PM, solid blue). Stimulation with ADP (2 μM at 50 s) lead to the increased maximal calcium concentration and integrin activation compared to the initial model (N, dashed curves). **(b)** Increased cytosolic calcium as a result of platelet pre-activation. Initial IP_3 concentration (not shown) was same as in HFB model on Fig. 3d due to increased PLC activity in the quiescent state; this lead to higher basal calcium concentration (solid red) without significant integrin activation (solid blue). Stimulation with CRP (1 μg/ml at 50 s) lead to higher maximal calcium concentration and integrin activation compared to initial model (N, dashed curves).

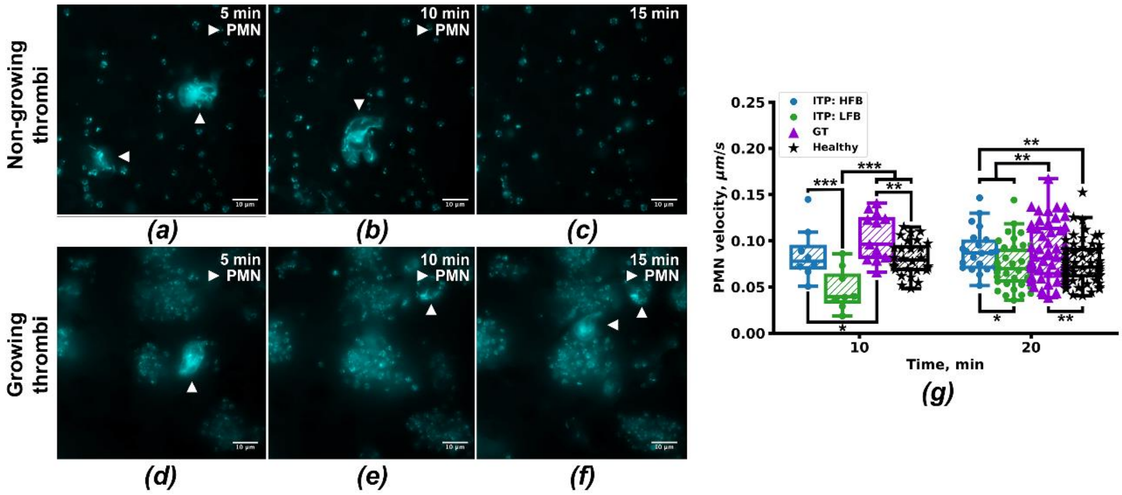

Figure S5. Thrombus growth kinetics. (a-c) Typical non-growing thrombi with the PMN crawling around them at 5 minutes (a), 10 minutes (b) and 15 minutes (c) after the start of the assay. (d-f) typical growing thrombi with the PMN crawling around them at 5 minutes (d), 10 minutes (e) and 15 minutes (f) after the start of the assay. Average PMN crawling velocity is significantly decreased in ITP: LFB group at 10 minutes, while GT patients had an increased PMN crawling velocity both at 10 and at 20 minutes.

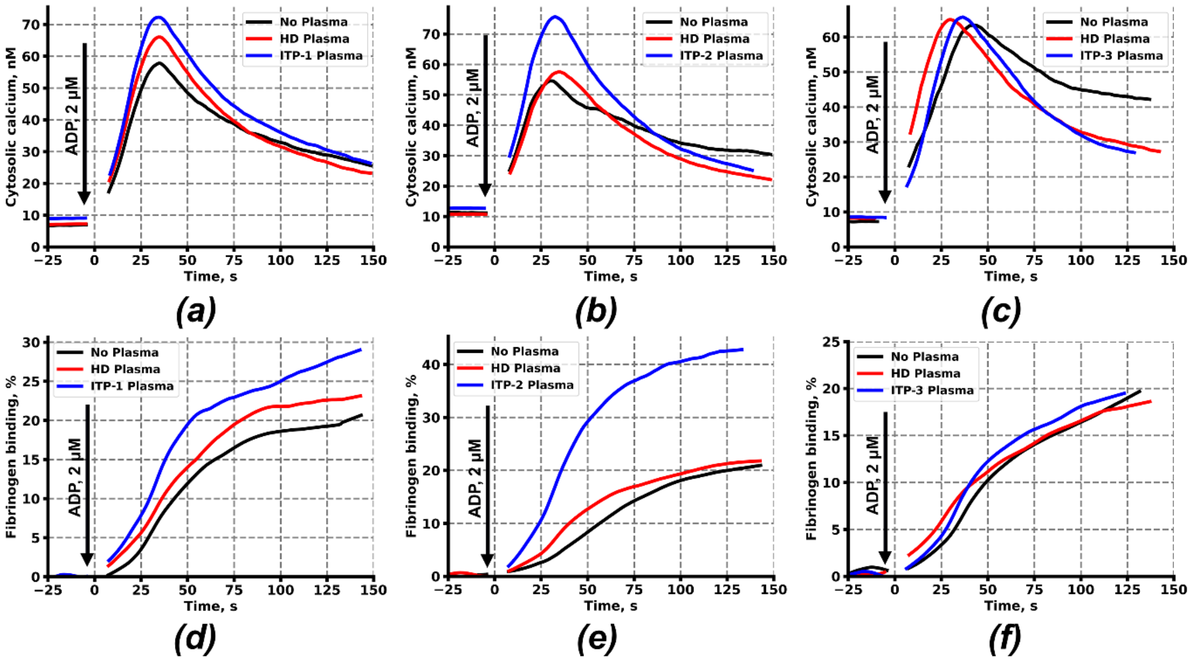

Figure S6. Impact of ITP plasma on healthy donor platelet reactivity. (a-c) Typical cytosolic calcium concentration and fibrinogen binding (d-f) responses upon activation by 2 μM of ADP after pre-incubation without healthy donor or ITP plasma (black curves), after pre-incubation with healthy donor plasma (red curves) or after pre-incubation with ITP (three different ITP patients) patient plasma (blue curves).

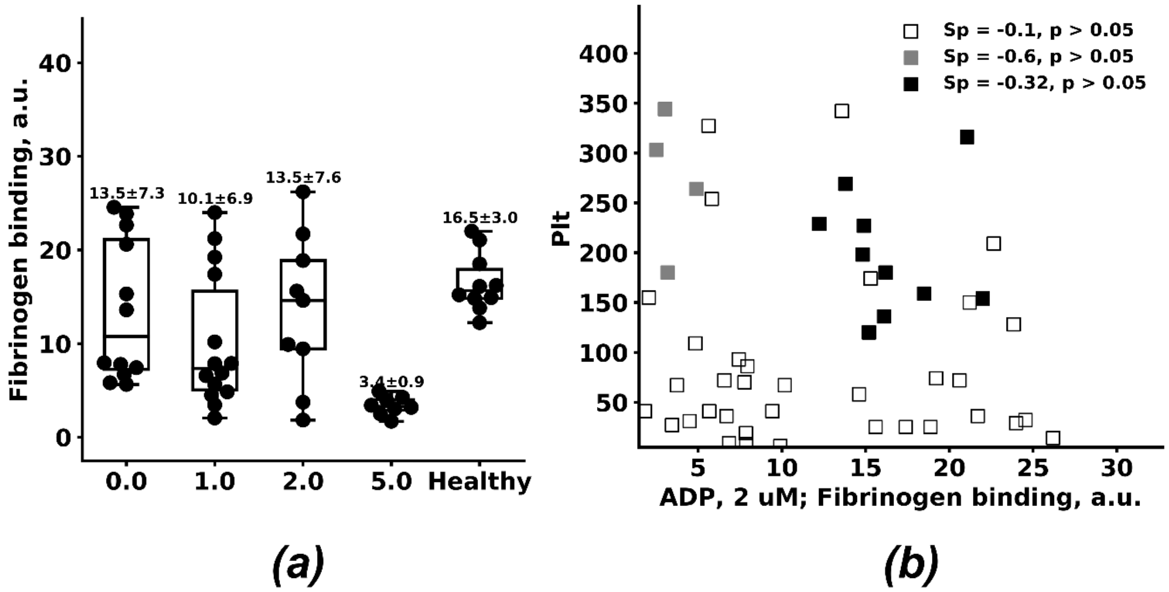

Figure S7. Fibrinogen binding in response to ADP as a function of patients' parameters.
Bleeding (a) and platelet count (b) as a function of fibrinogen binding in response to ADP, solid squares – healthy, grey squires – GT.

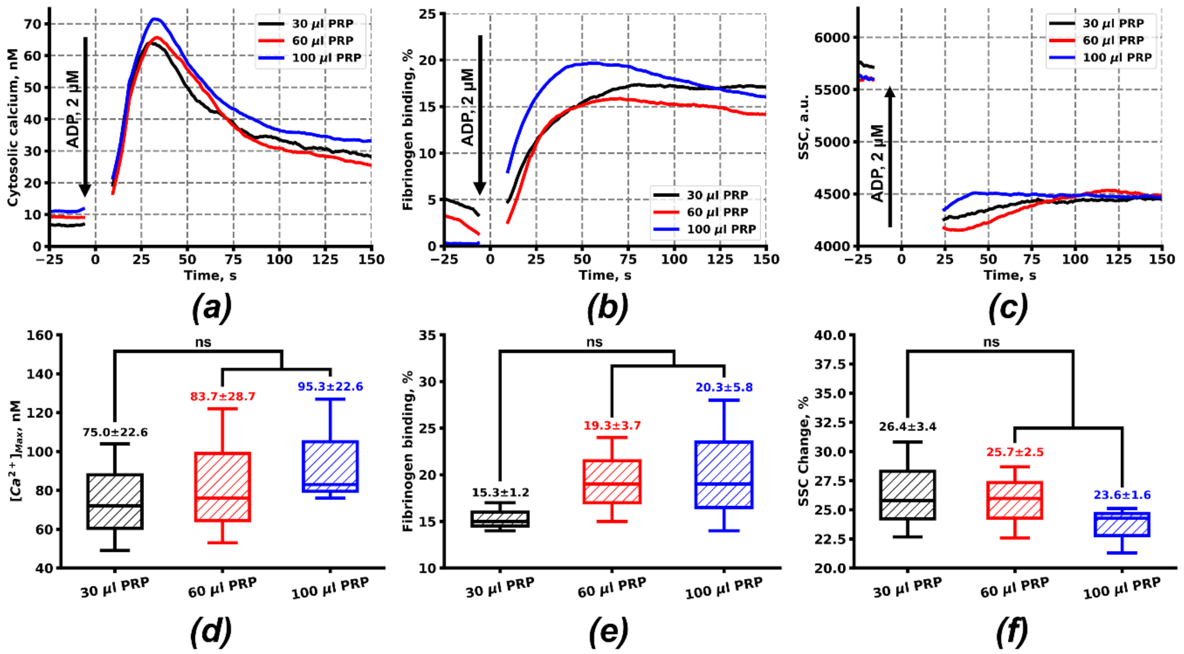

Figure S8. Impact of varying dilution of healthy donor LRP on platelet reactivity. (A-C) Typical cytosolic calcium concentration (a), fibrinogen binding (b), shape change (c) curves upon activation by 2 μM of ADP after 30 μl (black), 60 μl (red), 100 μl (blue) PRP dilution in tyrode's buffer. (d-f) No statistically significant changes in maximal calcium concentration (d), fibrinogen binding (e) and shape change (f) after activation by 2 μM ADP of differently diluted plasma was obtained ($n = 5$; ns: $p > 0.05$; statistical significance was calculated using Mann-Witney criteria).

Computational model development and analysis

The detailed model description with rate constants can be found in Table S2. The scheme of the reactions and signaling pathways incorporated into the model is given in Figure 3a. Activation of PAR1, PAR4, $P2Y_1$ and $P2Y_{12}$ was considered essentially as described previously [11–14]. Activation of GPVI and the corresponding signaling cascade was taken from our previous work [15]. The number of $P2Y_1$ receptors per platelet was assumed to be 200 [3]. The unknown parameters for the new reactions were adjusted based on the original experimental data (Fig. S9, see below). The model with fixed parameters was validated against experiments with activation of platelets with several receptors (Fig. S10, see below). Detailed descriptions of algorithms and strategies for parameter estimation were described earlier [12,16]. The set of ordinary differential equations was integrated using the COPASI software (http://www.copasi.org) [17]. For tuning the model, estimation of parameters and comparison between models the deterministic simulations were used (LSODE solver).

The system of ODEs for the integrin activation module was reduced to one equation to decrease the calculation time for the model. First, all the variables (Table S2) were made dimensionless with values in the order of 1 (*O(1)*). This procedure lead to the following system of ODEs:

$$\frac{dx_1}{dt} = 0.15 \cdot x_1 \cdot Ca \ + 0.01 \cdot x_2$$

$$\frac{dx_2}{dt} = +0.72 \cdot x_1 \cdot Ca \ - 0.048 \cdot x_2 \ - 0.072 \cdot x_2 \cdot Ca \ + 0.02 \cdot x_3$$

$$\frac{dx_3}{dt} = 0.36 \cdot x_2 \cdot Ca \ - 0.048 \cdot x_3$$

$$\frac{dx_4}{dt} = -0.0001 \cdot P3^{10} \cdot x_4 \ + 615 \cdot x_5$$

$$\frac{dx_5}{dt} = +0.0001 \cdot P3^{10} \cdot x_4 \ - 615 \cdot x_5$$

$$\frac{dx_6}{dt} = -0.18 \cdot x_3 \cdot x_6 \ + 0.0005 \cdot x_7 \ - 0.1 \cdot x_4 \cdot x_7 \ - 0.00015 \cdot x_6 \ + 0.075 \cdot x_8 \cdot [\mathrm{GDP}]$$

$$\frac{dx_7}{dt} = -\ (0.0005 \cdot x_7) \ - 0.1 \cdot x_4 \cdot x_7 \ - 0.00015 \cdot x_7 \ + 0.075 \cdot x_8 \cdot [\mathrm{GTP}]$$

$$\frac{dx_8}{dt} = +3.6 \cdot x_3 \cdot x_6 \ + 0.003 \cdot x_6 \ - 1.5 \cdot x_8 \cdot [\mathrm{GDP}] \ + 0.003 \cdot x_7 \ - 1.5 \cdot x_8 \cdot [\mathrm{GTP}]$$

$$\frac{dx_i}{dt} = -\frac{40 \cdot x_7^2}{4 \cdot x_7^2 + 0.2} \cdot x_i \ + 22.4 \cdot x_a$$

$$\frac{dx_a}{dt} = \frac{100 \cdot x_7^2}{4 \cdot x_7^2 + 0.2} \ - 56 \cdot x_a$$

where x_1 denotes inactive CalDAGGEFI, x_2 – half-active CalDAGGEFI, x_3 – active CalDAGGEFI, x_4 – active RASA3, x_5 – inactive RASA3, x_6 – Rap1-GDP, x_7 –Rap1-GTP, x_8 – Rap1, x_i – inactive integrins, x_a – active integrins, *P3* – PIP_3, *Ca* – free calcium ions in cytosol. The following reduction was based on the observation that *Ca* is the fast variable, while *P3* is more slow and $x_1 - x_8$ are even more slow. The final equation for integrin activation is:

$$\frac{d}{dt}[Rap1GTP] = \gamma \frac{[Rap1GDP][Ca_{cyt}^{2+}]^2}{K^2 + [Ca_{cyt}^{2+}]^2} - \frac{k}{1 + [G\beta\gamma]/l}[Rap1GTP],$$

where [Rap1GD(T)P] denotes the Rap1GD(T)P concentration, [Gβγ] – denotes Gβγ concentration. The parameters were adjusted to describe experimental data (Fig. S7) and were the following. γ = 0.035 1/s, K = 0.146 μM, *k* = 5.15 1/s, *l* = 1,8e-7 μM.

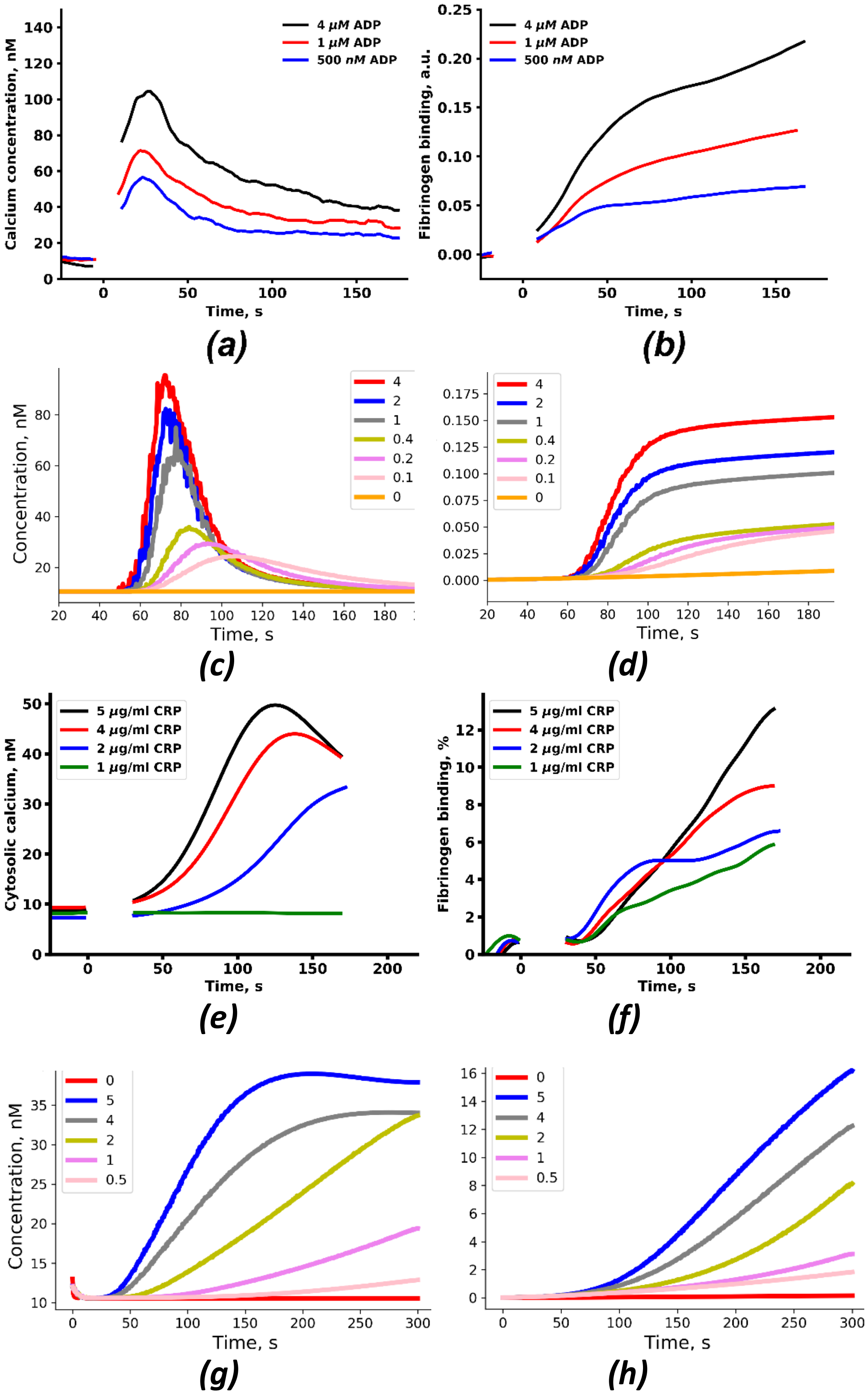

Figure S9. Parameter estimation for the integrin activation module of the computational model for healthy donors. (a,b,e,f) Continuous flow cytometry of the activation of platelets from healthy donors with ADP at 0.5-4 μM (a,b) and CRP at 1-5 μg/ml (e,f). (c,d,g,h) Deterministic simulation of the activation of a normal platelet with ADP at 0-4 μM (c,d) and CRP at 0-5 μg/ml (g,h) at 50 s. (a,c,e,g) Calcium concentration, nM; (b,d) integrin activation, fraction; (f,h) integrin activation, %.

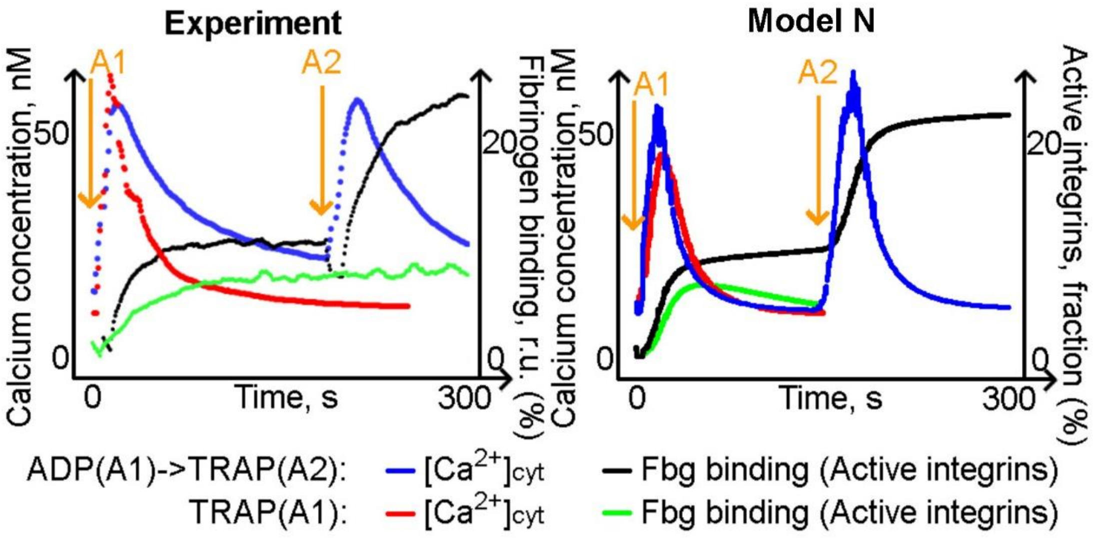

Figure S10. Validation of computational model. Validation of integrin activation module of the computational model for healthy donors. Left: continuous flow cytometry of the activation of platelets from healthy donors with TRAP at 1 μM or TRAP and ADP at 1 μM. Right: deterministic simulation of the activation of normal platelet with TRAP at 1 μM or TRAP and ADP at 1 μM.

References

1. Lee, S.B.; Shin, S.H.; Hepler, J.R.; Gilman, A.G.; Rhee, S.G. Activation of phospholipase C-beta 2 mutants by G protein alpha q and beta gamma subunits. *J.Biol.Chem.* **1993**, *268*, 25952–25957.
2. Philip, F.; Kadamur, G.; Silos, R.G.; Woodson, J.; Ross, E.M. Synergistic Activation of Phospholipase C-β3 by Gαq and Gβγ Describes a Simple Two-State Coincidence Detector. *Current Biology* **2010**, *20*, 1327–1335.
3. Burkhart, J.M.; Vaudel, M.; Gambaryan, S.; Radau, S.; Walter, U.; Martens, L.; Geiger, J.; Sickmann, A.; Zahedi, R.P. The first comprehensive and quantitative analysis of human platelet protein composition allows the comparative analysis of structural and functional pathways. *Blood* **2012**, *120*, e73–e82.
4. Domin, J.; Pages, F.; Volinia, S.; Rittenhouse, S.E.; Zvelebil, M.J.; Stein, R.C.; Waterfield, M.D. Cloning of a human phosphoinositide 3-kinase with a C2 domain that displays reduced sensitivity to the inhibitor wortmannin. *The Biochemical journal* **1997**, *326 (Pt 1)*, 139–47.
5. Maehama, T.; Dixon, J.E. The tumor suppressor, PTEN/MMAC1, dephosphorylates the lipid second messenger, phosphatidylinositol 3,4,5-trisphosphate. *The Journal of biological chemistry* **1998**, *273*, 13375–8.
6. Woscholski, R.; Waterfield, M.D.; Parker, P.J. Purification and biochemical characterization of a mammalian phosphatidylinositol 3,4,5-trisphosphate 5-phosphatase. *The Journal of biological chemistry* **1995**, *270*, 31001–7.
7. Tazmini, G.; Beaulieu, N.; Woo, A.; Zahedi, B.; Goulding, R.E.; Kay, R.J. Membrane localization of RasGRP1 is controlled by an EF-hand, and by the GEF domain. *Biochimica et Biophysica Acta (BBA) - Molecular Cell Research* **2009**, *1793*, 447–461.
8. Lewit-Bentley, A.; Réty, S. EF-hand calcium-binding proteins. *Current Opinion in Structural Biology* **2000**, *10*, 637–643.
9. Bourne, H.R.; Sanders, D.A.; McCormick, F. The GTPase superfamily: conserved structure and molecular mechanism. *Nature* **1991**, *349*, 117–127.
10. Jeyaraj, S.C.; Unger, N.T.; Chotani, M.A. Rap1 GTPases: an emerging role in the cardiovasculature. *Life sciences* **2011**, *88*, 645–52.
11. Sveshnikova, A.N.; Ataullakhanov, F.I.; Panteleev, M.A. Compartmentalized calcium signaling triggers subpopulation formation upon platelet activation through PAR1. *Molecular BioSystems* **2015**, *11*, 1052–1060.
12. Sveshnikova, A.N.; Balatskiy, A.V.; Demianova, A.S.; Shepelyuk, T.O.; Shakhidzhanov, S.S.; Balatskaya, M.N.; Pichugin, A.V.; Ataullakhanov, F.I.; Panteleev, M.A. Systems biology insights into the meaning of the platelet's dual-receptor thrombin signaling. *Journal of Thrombosis and Haemostasis* **2016**, *14*, 2045–2057.
13. Shakhidzhanov, S.S.; Shaturny, V.I.; Panteleev, M.A.; Sveshnikova, A.N. Modulation and pre-amplification of PAR1signaling by ADP acting via the P2Y12receptor during platelet subpopulation formation. *Biochimica et Biophysica Acta - General Subjects* **2015**, *1850*, 2518–2529.
14. Purvis, J.E.; Chatterjee, M.S.; Brass, L.F.; Diamond, S.L. A molecular signaling model of platelet phosphoinositide and calcium regulation during homeostasis and P2Y1 activation. *Blood* **2008**, *112*, 4069–4079.
15. Martyanov, A.; Balabin, F.A.; Dunster, J.L.; Panteleev, M.A.; Gibbins, J.; Sveshnikova, A.N. *Diffusional and chemical control in the tyrosine kinase network of platelet CLEC-2 signalling*; Biorxiv:529859, 2019;
16. Dunster, J.L.; Panteleev, M.A.; Gibbins, J.M.; Sveshnikova, A.N. Mathematical Techniques for Understanding Platelet Regulation and the Development of New Pharmacological Approaches. *Methods Mol. Biol.* **2018**, *1812*, 255–279.
17. Hoops, S.; Sahle, S.; Gauges, R.; Lee, C.; Pahle, J.; Simus, N.; Singhal, M.; Xu, L.; Mendes, P.; Kummer, U. COPASI–a COmplex PAthway SImulator. *Bioinformatics.* **2006**, *22*, 3067–3074.
